# Measuring direct and indirect tendon parameters to characterize the proximal tendinous complex of the rectus femoris in football and futsal players

**DOI:** 10.3389/fphys.2023.986872

**Published:** 2023-02-07

**Authors:** Sandra Mechó, Raquel Lisbona Ortega, Ricard Pruna, Lexa Nescolarde Selva, Jordi Morillas Pérez, Alfonso Rodríguez-Baeza, Javier Martínez Agea, Ricard Pérez-Andrés

**Affiliations:** ^1^ Autonomous University of Barcelona, Barcelona, Spain; ^2^ Department of Radiology Hospital de Barcelona, SCIAS, Barcelona, Spain; ^3^ Department of Radiology, Hospital Germans Trias i Pujol, Badalona, Spain; ^4^ FC Barcelona Medical Services, Sant Joan Despí, Spain; ^5^ Department of Electronic Engineering, Universitat Politècnica de Catalunya, Barcelona, Spain; ^6^ Unit of Intensive Care Medicine, Hospital de Barcelona, SCIAS, Barcelona, Spain; ^7^ Department of Morphological Sciences (Human Anatomy and Embryology Unit), Faculty of Medicine, Universitat Autònoma de Barcelona, Bellaterra, Spain

**Keywords:** MRI, rectus femoris, proximal tendinous complex, direct tendon and indirect tendon, DT-IT angle

## Abstract

**Objective:** To present unprecedented radiological parameters that characterize the angle between the direct and indirect tendons of the proximal rectus femoris (RF) and its inclinations and to evaluate the population variability according to demographic variables.

**Materials and methods:** From September 2019 to July 2021, using MRI multiplanar reconstructions of the proximal thigh/hip, two blinded radiologists measured the direct and indirect tendon angle and the inclination of each tendon in different planes. The intra- and inter-observer agreements were assessed with Bland–Altman analysis and intraclass correlation coefficient (ICC). The correlations between radiological parameters and demographic variables were evaluated using linear regression, Student’s *t-*test, and analysis of variance.

**Results:** We performed 112 thigh/hip MRI scans on 91 football players of different age, gender, and disciplines (football and futsal). For observer 1 (the reference), the mean direct and indirect tendon angle was 56.74° ± 9.37, the mean indirect tendon slope was −7.90° ± 7.49, and the mean direct tendon slope was 22.16° ± 5.88. The three measurements showed inter- and intra-observer agreement (mean differences ∼0). No correlation was observed between age and the parameters. Likewise, no statistically significant differences were found for gender, dominant limb, examined limb, and sport.

**Conclusion:** There is an inter- and intra-observer agreement in the measurements of the direct and indirect tendon angle and the inclination of each tendon. There is population variability in the proximal tendinous complex unrelated to demographic factors. These results allow further detection of morphological patterns that represent a risk factor for lesions in the RF in professional football and futsal players and other sports.

## Introduction

Muscle injury remains a diagnostic, therapeutic, and preventive challenge in sports medicine. More than a third of the injuries suffered by athletes annually are muscle injuries (Ekstrand J et al., 2011; Guermazi A et al., 2017; Isern-Kebschull J et al., 2020). Among quadriceps muscles, the rectus femoris (RF) is the most commonly injured and the third most common site of lower extremity muscle injury (Entwisle T et al., 2019). Indeed, a study on professional female football players characterized the quadriceps muscle complex as the most often injured (Larruskain et al., 2018).

The RF is a biarticular, fusiform muscle that tends to carry out eccentric contractions with a high percentage of fast-twitch (type II) fibers. This explains why the RF is the quadriceps muscle more frequently injured in repetitive kicking and sprinting sports (Hughes C et al., 1995; Morelli V and Weaver V., 2005; Kassarjian A et al., 2012; Mendiguchia J et al., 2013; Mariluis and CupitoMamone, 2015; Entwisle T et al., 2019).

The myotendinous junction (MTJ) of the central septum is the most common site of RF injury in football (Balius R et al., 2009; Mendiguchia J et al., 2013; Mariluis and CupitoMamone, 2015) and is associated, particularly proximal injuries, with long rehabilitation and delayed return-to-play times (Cross TM et al., 2004; Ochard J et al., 2005; Balius R et al., 2009; Mendiguchia J et al., 2013; Brukner P and Connell D, 2015). The central septum belongs to the proximal MTJ of the RF. As its origin is complex, the term proximal tendon complex (PTC) will be applied from here onward. The PTC consists of two tendons: the direct or anterior tendon (DT) and the indirect or reflected tendon (IT). Sometimes, there is a third tendon, originating in the IT and attached to the structures that delimit it (Tubbs RS, 2006). Ultimately, these tendons converge into a conjoined tendon (CT). The tendinous origin of the RF has been widely described in the medical literature (Renstrom PA, 1992; Hasselman CT et al., 1995; Bianchi S et al., 2002; Bordalo-Rodrigues M and Rosenberg ZS, 2005; Ouellette H et al., 2006; Tubbs RS, 2006; Gyftopoulos S et al., 2008; Balius R et al., 2009; Gamradt SC et al., 2009; Kassarjian A et al., 2012; Ryan JM et al., 2014; Mariluis and CupitoMamone, 2015; Moraux A et al., 2015), but the angle formed by the heads to converge on the CT and their inclination have not been previously studied. Considering all the aforementioned factors, a detailed characterization of all elements forming the proximal MTJ of the RF and their relationship would be of interest to detect possible patterns with a high risk of injury and to reinforce injury prevention.

Therefore, this study aims to present unprecedented radiological parameters to characterize the direction of the DT and IT tendons when converging on the CT, in the sagittal plane (i.e., the DT-IT angle), and to characterize the inclinations of the DT and IT in different planes. It also aims to evaluate the population variability of these PTC structures and their variability according to demographic factors.

## Materials and methods

### Patients

The Clinical Research Ethics Committee of the Sports Administration of Catalonia (Spain) approved this prospective study. Written informed consent was obtained from all participants included in this study.

From September 2019 to July 2021, 100 patients or volunteers were initially included to perform hip or proximal thigh MR examinations with a 3D PD sequence added to the standard protocol. All participants were football players of different disciplines (association football and futsal) and categories (junior, young, and professional teams).

**FIGURE 1:**
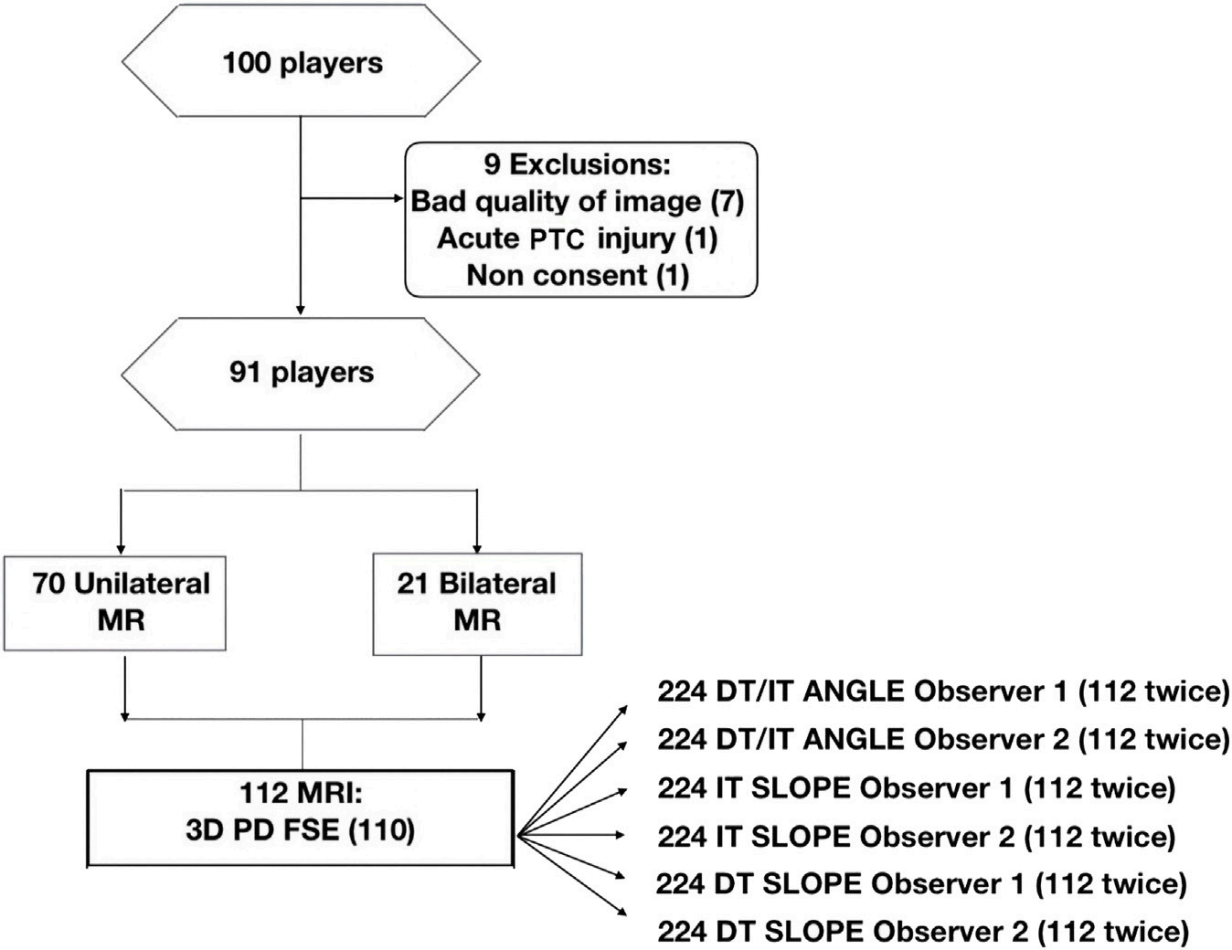
Flowchart of the participants in the study. DT, direct tendon; FSE, fast spin echo; IT, indirect tendon; PD, proton density; PTC, proximal tendon complex.

Patients with a clinical indication, some volunteers who willingly participated in the study and could understand the reason behind it, and those who had signed informed consent were included for a hip or proximal thigh MR. We excluded seven cases where artifacts or movement caused an inhomogeneity of the field under study, one case with a poor definition of tendon structures due to acute tendon injury, and one case who did not consent to participate in the study (Figure 1).

Each patient’s demographic data were recorded (age, gender, football discipline, lateral side, dominance, examination results, reason for examination, and history or acute injury of the proximal MTJ injury of the RF) in the database.

### Image acquisition

The explorations were performed using a Canon Medical Vantage Titan 3 T MR scanner, with a maximum gradient strength of 45 mT/m, 203 T/m/s slew rate, and 32 receiver channels. An axial sequence was added to the standard thigh or hip protocol consisting of a 3D proton density (PD) contrast; TR 5200 ms; TE 44–60 ms; echo train spacing 7.5; slice thickness 2.5–3.5 mm; in-plane resolution 1.4 × 0.88 mm^2^; and FOV 256 × 256 mm. The examination duration for this sequence was 8 min. This sequence provides datasets that can be retrospectively post-processed for viewing in freely selectable orientations, thus being advantageous for evaluating the complex anatomy associated with the PTC (Mugler, 2014).

The subjects were placed in a supine position, with the limb covered by a 16-element phased array coil (Body). Twenty elements of the table-integrated coil were also used, combined in a total of 32 independent receiver channels. The feet were fixed in a neutral position using a strap to standardize and fix the position of the extremities, and the symmetrical horizontal position of the femoral heads was verified using an MR pelvis locator scan. Totally, 172 axial images of the proximal portion of the thigh were obtained from the lower margin of the anterior superior iliac spine to the origin of the proximal anterior myoaponeurotic junction of the RF.

The images were post-processed with the validated software Vitrea, and a multiplanar reconstruction was obtained, which allowed new oblique planes oriented to the anatomy of both tendons. 3D sequences were used for multiplanar reconstructions to avoid obtaining oblique sequences with post-processing artifacts.

### DT-IT angle plane

**FIGURE 2:**
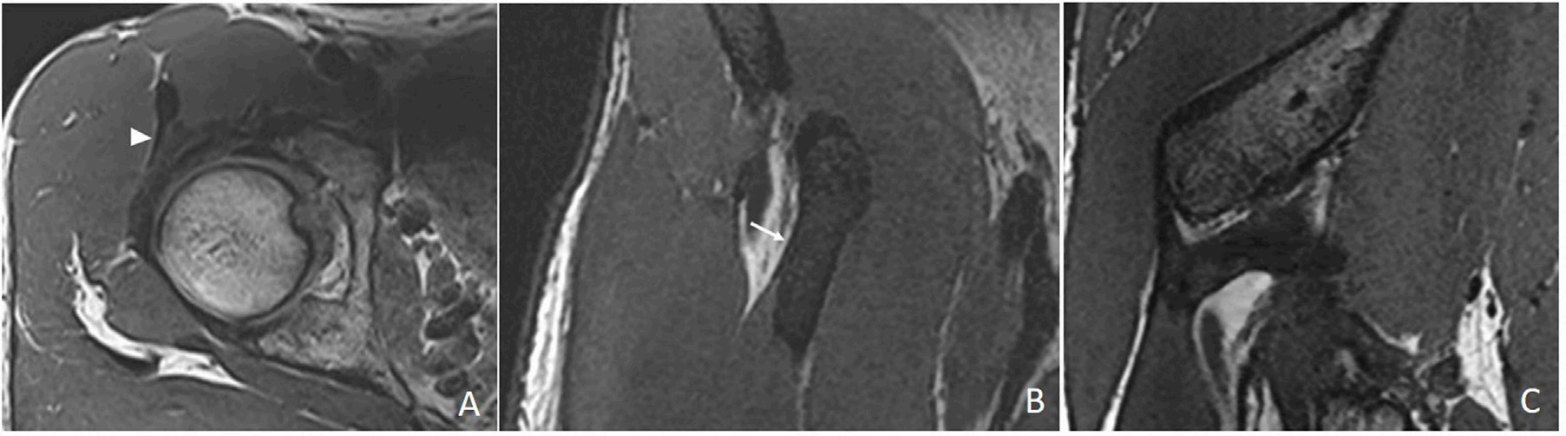
Multiplanar reconstruction. **(A)** Chosen axial 3D proton density section to orientate the sagittal plane following the lateral margin of the anterior portion of the indirect tendon (white arrowhead). **(B)** Chosen coronal 3D proton density section to orientate the sagittal plane following the lateral margin of the direct tendon (white arrow). **(C)** Resulting oblique sagittal plane.

The DT-IT angle was measured in an oblique sagittal plane obtained by multiplanar reconstruction based on the orientation of the IT axis in the axial plane and the DT axis in the coronal plane (Figure 2).

The section where the IT was thickest in a greater length in the axial plane was chosen. The inclination was oriented, taking the most lateral margin of the anterior portion of the tendon as a reference (Figure 2A). In the coronal plane, the section was selected where the transverse diameter of the DT was greatest. The inclination was oriented with respect to the most lateral margin of the DT (Figure 2B).

### IT slope plane

**FIGURE 3:**
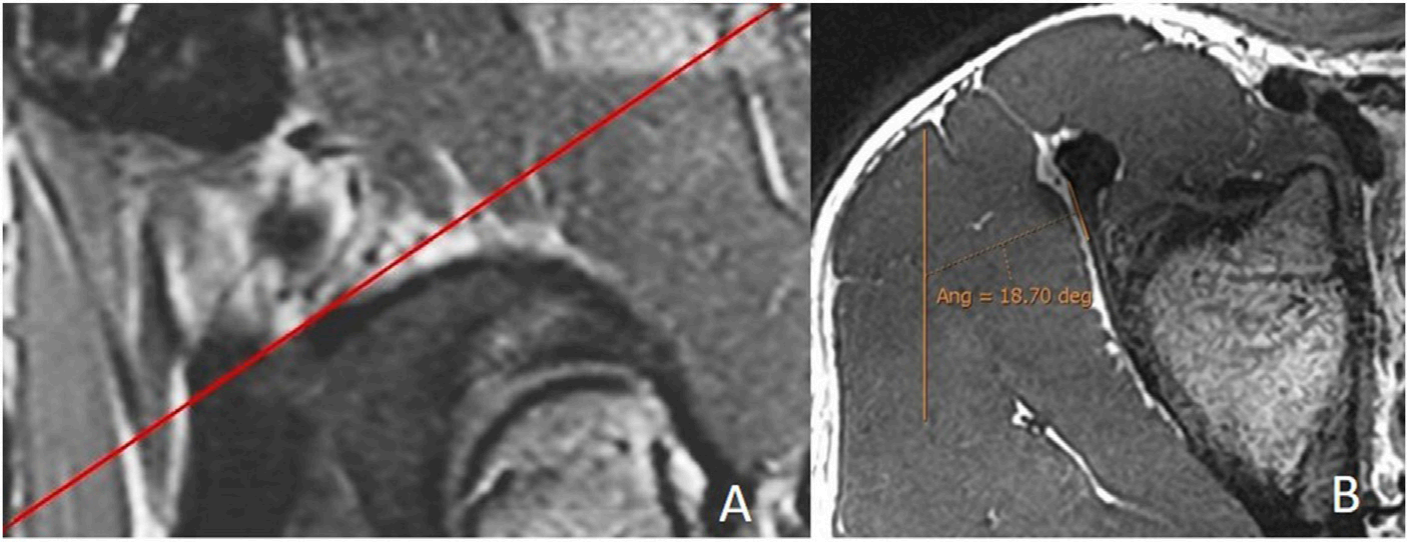
Measurement of the indirect tendon slope. **(A)** Sagittal 3D proton density with the reference line to obtain the batch of the oblique axial reconstruction following the orientation of the indirect tendon. **(B)** Median section of the oblique axial reconstruction of the indirect tendon with measurement of its inclination (indirect tendon anterolateral margin/anteroposterior axis). The portion of the CT is excluded. In this case, the merging of the indirect tendon with the CT in the axial plane is more lateral than the acetabular insertion of the indirect tendon, and the inclination is negative (−18.7°).

On the sagittal images, the plane in which the junction of the IT with the CT was the clearest was chosen, and an oblique axial plane was obtained after the IT orientation in the portion closest to the CT (Figure 3).

### DT slope plane

The DT slope was obtained using a coronal plane, selecting the plane with the greatest transverse diameter of the tendon. The inclination was oriented with respect to the most lateral margin of the DT.

### Measurements

The obtained reconstructions were exported in batch format to PACS to perform measurements. The reconstructions and the 3D PD axial sequence with Alma 2D Dicom Viewer were analyzed using a layout of different views (usually four). The three desired angles (DT-IT angle, IT slope, and DT slope) were obtained with the “Angle with Baseline” tool.

**FIGURE 4:**
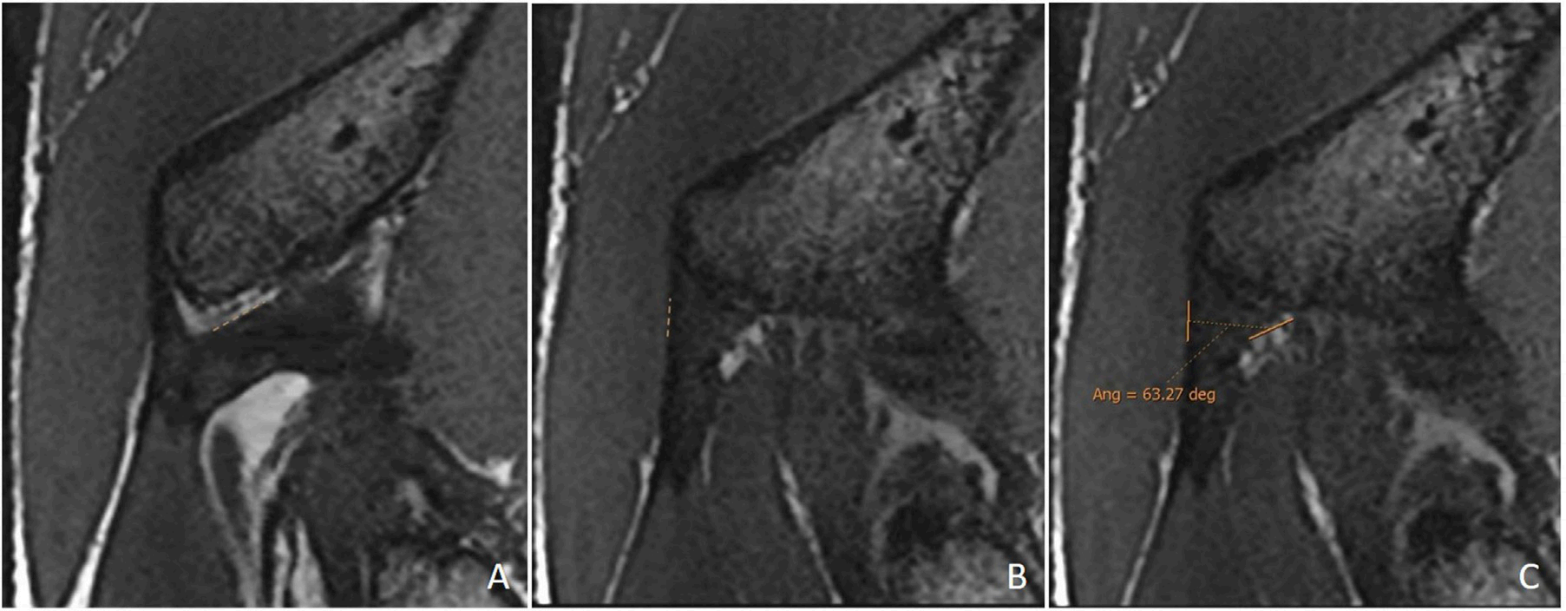
Double oblique sagittal 3D proton density sections. **(A)** Lateral section of the indirect tendon with the cranial margin of the IT (indirect tendon line of the angle). **(B)** Middle line section of the direct tendon with the anterior margin of the tendon draughted (direct tendon line of the angle) and **(C)** resulting direct–indirect tendon angle.

Double obliquity sagittal reconstruction was used to measure the DT-IT angle (Figure 4). The first lateral section was chosen to obtain the IT component, where its upper margin was clearest (hypointense fine line), and the line delimiting this margin was drawn (Figure 4A). Only the portion of the free tendon was considered (tendinous portion without insertional expansions to the acetabular sulcus and joint capsule). The section corresponding to the middle third of the tendon was chosen to delimit the DT line, considering the coronal image as a reference. The line delimiting the anterior margin of the DT was drawn (Figure 4B).

Oblique axial reconstruction was used to measure the IT slope. In the middle section of the IT, the inclination of this tendon was measured with respect to the anteroposterior axis, tracing the line of the IT on the most lateral margin of the free tendinous portion. A negative value was considered when, in the axial plane, the junction of the IT with the CT was located in a more lateral location than the acetabular insertion of the IT (Figure 3).

**FIGURE 5:**
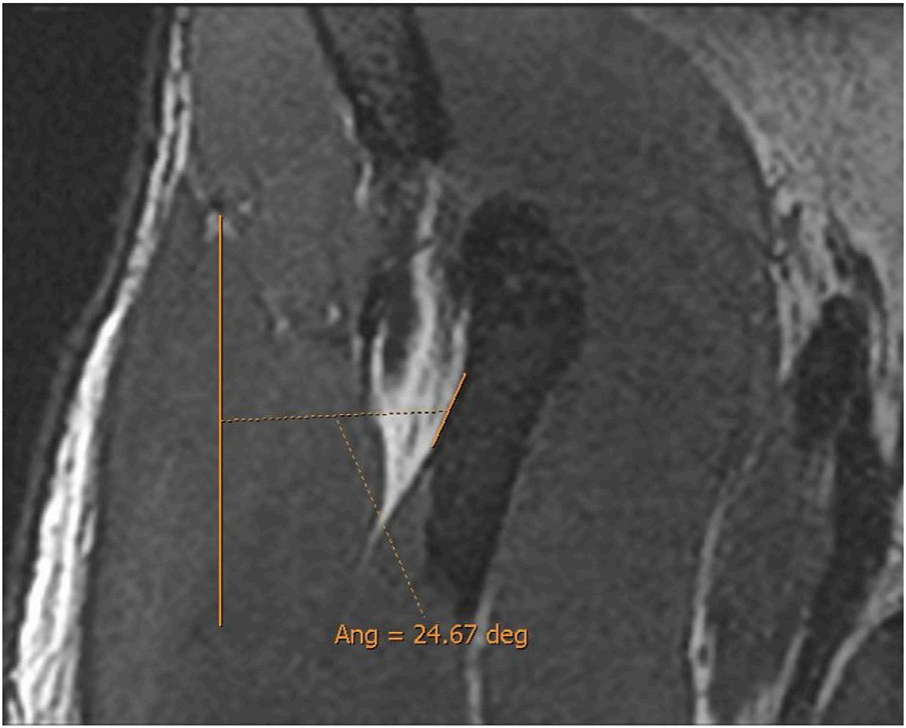
Oblique coronal 3D proton density image. Measurement of the direct tendon slope (direct tendon lateral margin/craniocaudal axis).

Coronal reconstruction was used to measure the DT slope. In the middle section of the DT, the inclination of this tendon was measured with respect to the craniocaudal axis, considering the lateral margin of the tendon as a reference point (Figure 5).

Post-processing of the obtained images was conducted by two radiologists specialized in musculoskeletal imaging with 13 (SM) and 2 (RL) years of experience. Both radiologists conducted a previous 3-month work to agree on the most appropriate measurement methods. Each radiologist assessed the images, performed the post-processing, and measured the DT-IT angle and the slopes independently (they were blind to the affiliation and clinical data of patients and the results of each other). To assess the intra-observer agreement of the measurement method, each radiologist undertook this measurement process twice, using the same images. In cases of disagreement, the causes were analyzed (most were due to poor definition of the tendon margins), and the methodology was revised. The intra-observer agreement was evaluated by means of a second randomized reading, with an interval between 4 and 12 months (a mean of 8 months).

### Data analysis

The categorical variables are shown as absolute frequencies and percentages.

The normality of distribution in the continuous variables was determined by the Kolmogorov–Smirnov test (*n* = 112). The normally distributed variables are shown as mean ± SD, 95% confidence interval for mean (lower and upper bound), whereas the non-normally distributed data are shown as median (interquartile range, IQR) and minimum–maximum.

The Bland–Altman plot (Bland JM and Altman DG, 1999; Giavarina D, 2015) was used to evaluate mean biases and 95% limit of inter- and intra-observer agreements. In the case of the inter-observer analysis, observer 1 was taken as a reference, and the analysis was made between the first measure of each observer. The B&A plot difference percentage was also performed. This is useful when there is an increase in variability of the differences as the magnitude of measurements increases (Bland JM and Altman DG, 1999). The sample size for the Bland–Altman plot was estimated for Type I error (α, significance) of 0.01 and Type II error (β, 1-Power) of 0.20 using MedCalc statistical software version 19.0.3 (MedCalc Software bvba, Ostend, Belgium).

The intraclass correlation coefficient (ICC) was used to study the intra- and inter-observer reliability for the measurement of the radiological parameters (DT-IT angle, IT slope, and DT slope). The data were analyzed using a mean of 112 measurements, absolute agreement, and a two-way mixed-effects model (Koo and Li, 2016).

One-way ANOVA test and Student’s *t-*test were used to determine statistical differences in the radiological parameters (DT-IT angle, IT slope, and DT slope), considering gender (male and female), football discipline (futsal and association football), lateral side (right and left), dominance (right and left), and age (<18 y and ≥18 y). Linear regression analysis was also used to determine statistical differences in the radiological parameters considering age**.**

The statistical software IBM^®^ SPSS^®^ version 26.0 (Armonk, NY: IBM Corp, United States) was used for data analysis. The level of statistical significance was set at *p* < 0.05.

## Results

Post-processing and measurement of 112 examinations of 91 players were performed (Figure 1).

The clinical and demographic characteristics of the participants are described in Table 1. The mean age was 21 (range 12–33) years. Dividing the age into ranges resulted in two categories: 81 participants older than 18 and 31 participants younger than 18 . Most participants were football players (83, 91.2%), and the dominant limb was examined in 66 (58.9%) cases. Most examinations were performed on the proximal thigh (105, 93.8%) for injuries in the hamstrings (45, 40.2%), the RF (26, 23.2%), or the adductor (18, 16.1%). In 35 cases (31.25%), there was a history of proximal MTJ injury of the RF, or an acute injury was identified in this location on the examination.

**TABLE 1:** Baseline demographic and epidemiological data of the studied population.

Gender	
Female	13 (14.3)
Male	78 (85.7)
Age (years), *mean* (range)	21 (12–33)
<18 years	31 (27.7)
≥18 years	81 (72.3)
Laterality	
Right limb	58 (51.8)
Left limb	54 (48.2)
Dominant limb examined	66 (58.9)
Sport	
Association football	83 (91.2)
Futsal	8 (8.8)
Body part examined	
Hip	7 (6.2)
Thigh	105 (93.8)
Reason for exploration (injured body part)	
Hamstrings	45 (40.2)
Rectus femoris	26 (23.2)
Adductors	18 (16.1)
Femoroacetabular impingement	4 (3.6)
Psoas iliacus	4 (3.6)
Contract signing exploration	4 (3.6)
Volunteer	4 (3.6)
Quadriceps	2 (1.8)
Other	5 (4.5)
Figures are absolute numbers (and %) unless otherwise stated	

The descriptions of radiological measurements for both observers are shown in Table 2. All the measurements are normally distributed variables, except for the second measurement of the IT slope of observer 2. The mean of the first measure of the DT-IT angle of both observers was similar (∼57°). The mean of the first measure of the IT slope of both observers was similar (∼−7.5°). The mean of the first measure of the DT slope of both observers was similar (∼22°). In all three cases, the mean of the second measure of both observers was similar to that of the first measure.

**TABLE 2:** Descriptions of radiological measurements of DT-IT angle, IT slope, and DT slope for observer 1 and observer 2.

Radiological measurements (grades)	Observer 1		Observer 2	
	First measure	Second measure	First measure	Second measure
Direct tendon–indirect tendon angle	56.74 ± 9.37 (54.98–58.49)	57.05 ± 9.49 (55.28–58.83)	57.13 ± 9.75 (55.30–58.95)	57.37 ± 9.83 (55.53–59.21)
Indirect tendon slope	−7.90 ± 7.49 (−9.30–(−6.50))	−7.33 ± 7.44 (−8.72–(−5.93))	−7.98 ± 7.69 (−9.42–(−6.54))	−7.60 (11.85) −26.93–11.51
Direct tendon slope	22.16 ± 5.88 (21.06–23.26)	22.36 ± 5.66 (21.30–23.42)	22.22 ± 5.88 (21.12–23.33)	22.27 ± 6.00 (21.15–23.39)

The variables normally distributed are shown as mean ± SD, 95% confidence interval for mean (lower bound and upper bound), whereas non-normally distributed data are shown as statistic median (interquartile range, IQR) and minimum–maximum.

Considering the sample calculation for Bland–Altman (B&A) analysis, the maximum required number of pairs for the angle DT-IT measurement was 18 cases; for the IT slope, 11 cases; and for the DT slope, 15 cases.

**FIGURE 6:**
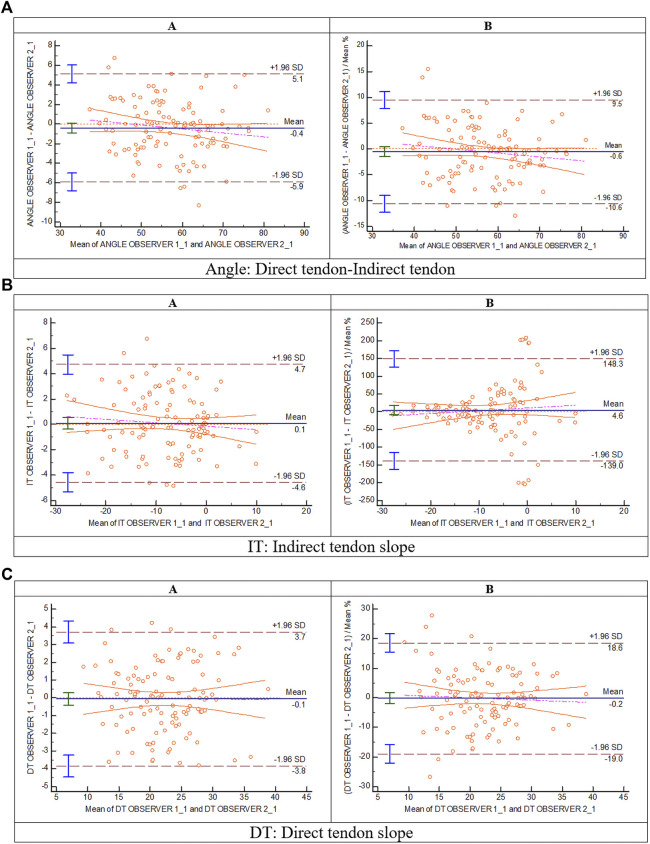
Bland and Altman: inter-observer analysis (A: Bland and Altman plot; **(B)** plot of differences between observer 1 and observer 2 as percentages of the values on the axis *vs*. the mean of the two measurements) for **(A)** direct tendon–indirect tendon angle, **(B)** indirect tendon slope, and **(C)** direct tendon slope.

**FIGURE 7:**
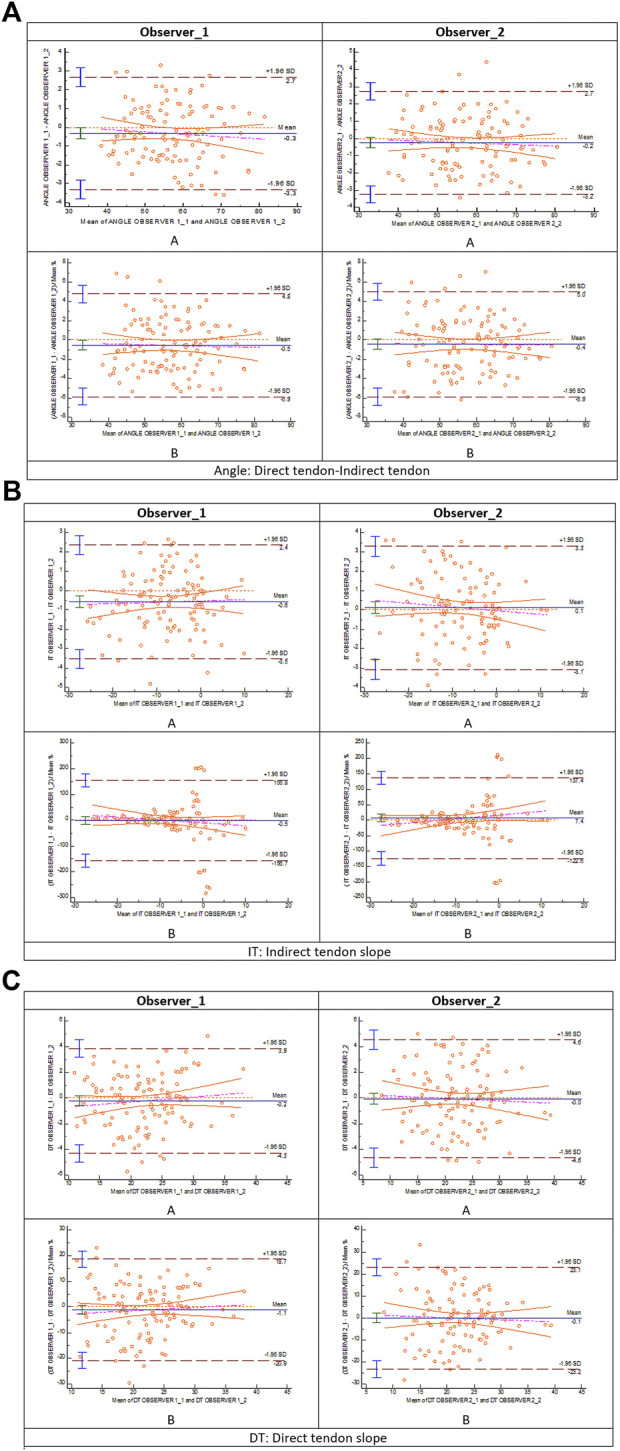
Bland and Altman: intra-observer analysis (A: Bland–Altman plot; **(B)** plot of differences between measure 1 and measure 2 for observer 1 and observer 2 as percentages of the values on the axis *vs*. the mean of the two measurements) for **(A)** direct tendon–indirect tendon angle, **(B)** indirect tendon slope, and **(C)** direct tendon slope.

The inter-observer agreement B&A analysis for the different radiological measurements is represented in Figure 6. The mean difference of the first measure between observer 1 and observer 2 for angle DT-IT (Figure 6A), IT slope (Figure 6B), and DT slope (Figure 6C) was ∼0 grade. The intra-observer agreement B&A analysis for the different radiological measurements is represented in Figure 7. In the case of the angle DT-IT and DT slope measurement (Figures 7A, C), the intra-observer agreement was similar for both observers, showing values of ∼ −0.2 and ∼ −0.1, and 95% of the data points lie within ± 2 s of the mean difference. In the case of the IT slope measure of observer 1, the intra-observer agreement B&A analysis showed that 0 is not included in the confidence interval limits for mean difference (Figure 7B). However, the mean difference is close to 0 (−0.6).

The ICC results are described in Table 3, showing reproducibility (with a tendency to absolute concordance) of the measurement method. The ICC value for all cases was greater than 0.9. The case with less correlation was the intra-observer 2 DT slope measurement, in which the ICC value was 0.92 with a 95% confidence interval of 0.888–0.946.

**TABLE 3:** ICC intra- and inter-observer analysis of the DT-IT angle, IT slope, and DT slope measurements, including intraclass correlation coefficient (ICC) and 95% confidence interval (CI).

Observer 1							
	95% CI			*F* test with true value 0			
	ICC	Lower bound	Upper bound	F-value	*df*1	*df*2	*p-*value^a^
DT-IT angle	0.987	0.981	0.991	152.059	111	111	0.00
IT slope	0.98	0.971	0.986	97.878	111	111	0.00
DT slope	0.934	0.906	0.954	29.477	111	111	0.00

Observer 2							
DT-IT angle	0.988	0.982	0.992	163.440	111	111	0.00
IT slope	0.978	0.968	0.985	90.203	111	111	0.00
DT slope	0.922	0.888	0.946	24.586	111	111	0.00

**Observer 1 *vs*. Observer 2**							
	95% CI			*F* test with true value 0			
	ICC	Lower bound	Upper bound	F-value	*df*1	*df*2	*p-*value^a^
DT-IT angle	0.957	0.938	0.97	45.42	111	111	0.00
IT slope	0.951	0.93	0.966	39.951	111	111	0.00
DT slope	0.946	0.923	0.963	36.326	111	111	0.00

DT, direct tendon; IT, indirect tendon.

^a^ Statistical significance of ICC analysis.

**FIGURE 8:**
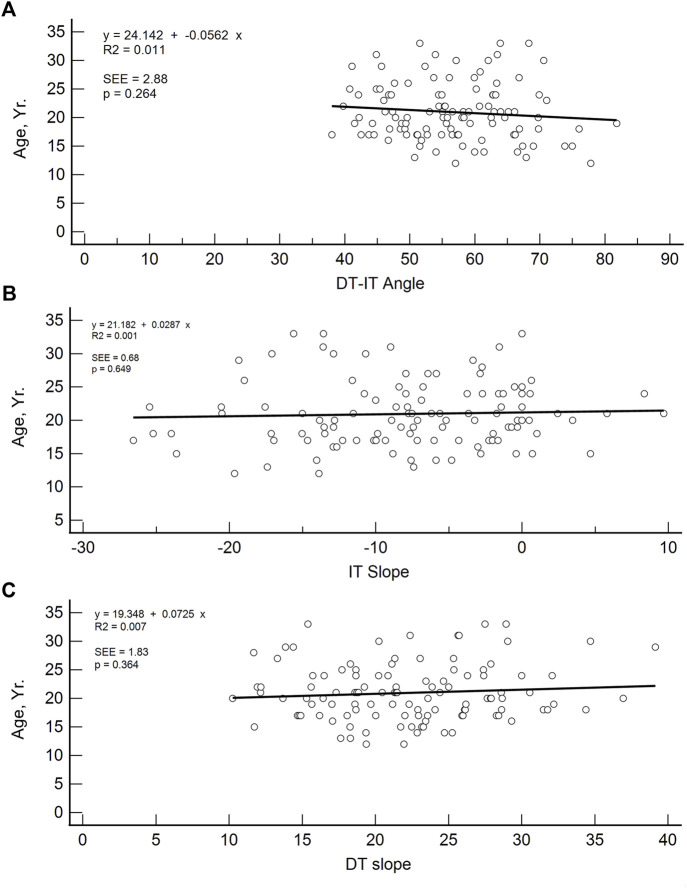
Linear regression plots of DT-IT angle **(A)**, IT slope **(B),** and DT slope **(C)** with respect to age (y).

Likewise, the results of Student’s *t-*test and one-way ANOVA test showed no statistically significant differences in the measured DT-IT angle and tendon inclinations for the means of the distributions corresponding to gender, dominant side, examined limb, and football discipline (Tables 4, 5). However, the Student’s *t*-test (Table 6) showed a correlation between the DT slope and the age range, and the older group showed a steeper slope. Conversely, linear regression analysis in Figure 8 showed that the participant’s age and the measured DT-IT angle or tendon inclinations (i.e., IT and DT slopes) were not significantly correlated.

**TABLE 4:** Statistical Student’s *t-*test correlation of the DT-IT angle, IT slope, and DT slope measured by observer 1 with demographic factors, including 95% confidence interval (CI).

DT-IT angle					
95% CI					
	Mean differences	Standard error differences	Lower bound	Upper bound	*p-*value^a^
Gender	0.003	2.69	−5.3	5.3	0.84
Examined limb	2.4	1.77	−1.09	5.9	0.9
Dominant limb	−0.15	2.16	−4.4	4.7	0.94
Sport	−3.5	3.1	−9.7	2.6	0.69

IT slope					
Gender	−1.08	2.15	−5.34	3.17	0.62
Examined limb	1.9	1.4	−0.86	4.73	0.43
Dominant limb	−0.98	1.13	−1.37	3.34	0.39
Sport	1.36	2.5	−3.57	6.3	0.55

DT slope					
Gender	2.45	1.67	−0.86	5.76	0.44
Examined limb	−1.5	1.12.35	−3.7	0.68	0.38
Dominant limb	−0.47	1.22	−2.08	3.02	0.7
Sport	−2.5	1.94	−6.3	1.4	0.78

DT, direct tendon; IT, indirect tendon.

a Statistical significance of Student’s *t*-test.

**TABLE 5:** One-way ANOVA test of the DT-IT angle, IT slope, and DT slope measured by observer 1 depending on the demographic factors, including 95% confidence interval (CI) and *F*-Fisher.

DT-IT angle					
95% CI					
	Mean ± SD	Lower bound	Upper bound	*F*	*p-*value^a^
Gender					
Male	56.74 ± 9.4	54.85	58.62	0	0.99
Female	56.73 ± 9.4	51.74	61.72		
Examined limb					
Right	57.89 ± 9.2	55.46	60.32	1.85	0.18
Left	55.49 ± 9.5	52.97	58		
Dominant limb					
Dominant	55.06 ± 8.98	51.21	58.91	0.003	0.95
Non-dominant	55.21 ± 8.47	51.36	59.06		
Sport					
Football	56.42 ± 9.3	54.58	58.25	1.31	0.25
Futsal	59.97 ± 9.9	54.1	65.84		

IT slope					
Gender					
Male	−8.04 ± 7.5	−9.54	−6.53	0.25	0.6
Female	−6.95 ± 7.62	−10.93	−2.97		
Examined limb					
Right	−6.97 ± 8.01	−8.91	−5.03	1.88	0.17
Left	−8.9 ± 6.8	−10.9	−6.89		
Dominant limb					
Dominant	−7.18 ± 7.44	−10.14	−4.21	0.22	0.64
Non-dominant	−6.19 ± 5.89	−9.15	−3.23		
Sport					
Football	−7.78 ± 7.34	−9.25	−6.3	0.3	0.58
Futsal	−9.14 ± 9.23	−13.85	−4.44		

DT slope					
Gender					
Male	22.46 ± 5.98	21.29	23.63	2.15	0.14
Female	20.01 ± 4.74	16.91	23.11		
Examined limb					
Right	21.43 ± 6.02	19.9	22.95	1.88	0.17
Left	22.94 ± 5.67	21.36	24.52		
Dominant limb					
Dominant	22.11 ± 6.41	19.68	24.54	0.08	0.78
Non-dominant	22.58 ± 4.45	20.15	25.02		
Sport					
Football	21.94 ± 5.82	20.79	23.09	1.6	0.21
Futsal	24.4 ± 6.34	20.72	28.07		

DT, direct tendon; IT, indirect tendon.

^a^ Statistical significance of one-way ANOVA test.

**TABLE 6:** Student’s *t*-test analysis of the DT-IT angle, IT slope, and DT slope measured by observer 1 depending on the age ranges (≥18 y and <18 y).

Student’s *t-*test						
95% CI						
	Mean ± SD	Mean differences	SE differences	Lower Bound	Upper Bound	*p-*value^a^
DT-IT angle						
≥18	56.46 ± 9.19	−1.01	1.99	−4.94	2.93	0.61
<18	57.46 ± 9.95					
IT slope						
≥18	−7.4 ± 7.60	1.81	1.58	−1.32	4.94	0.64
<18	−9.2 ± 7.10					
DT slope						
≥18	22.61 ± 6.29	1.62	1.24	−0.83	4,08	0.04
<18	20.98 ± 4.52					

DT, direct tendon; IT, indirect tendon.

^a^ Statistical significance of Student’s *t*-test.

The population available in the study is small to be able to assess the correlation between the radiological parameters and the risk of proximal MTJ injury of the RF. However, a small sample of three players was taken as an example, considering the history or presence of RF injury [two players with a history or acute injury (case B-C) and one player with no history of injury (case A)]. It could be seen that high values of the DT-IT angle (77.84°, 63.27°) and rectification of the inclinations of the IT (−1.41°) and the DT (16.14°) were characteristics of the injured cases. Therefore, they can be considered risk characteristics (Table 7).

**TABLE 7:** DT-IT angle, IT slope, and DT slope in one male football player with no rectus femoris injury and two male football players with different rectus femoris injuries. Bold values are outliers. DT, direct tendon; IT, indirect tendon.

Type of injury	Angle DT-IT	IT slope	DT slope	Injury figures
**Case A** (no rectus femoris injury)	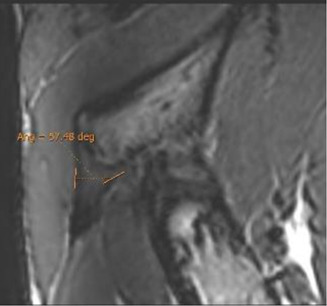 57.46°	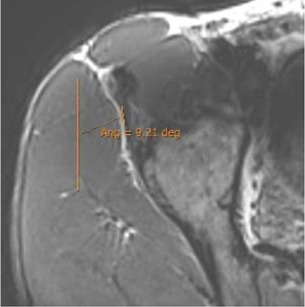 −9.21°	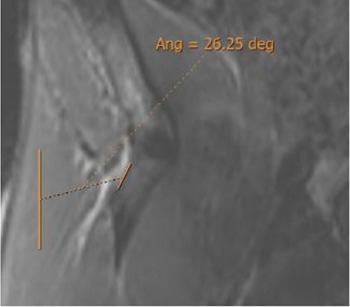 26.25°	
**Case B** Paraseptal acute and previous muscle injuries Scar of previous paraseptal muscle injury (arrowhead) and interstitial edema of paraseptal acute injury (arrow)	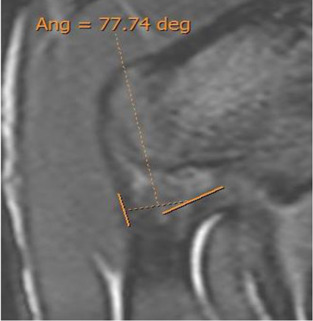 **77.74**°	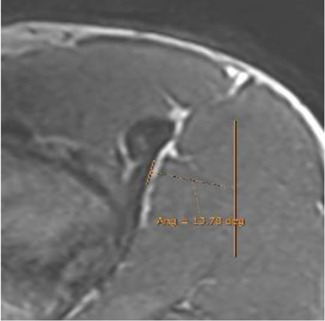 −13.73°	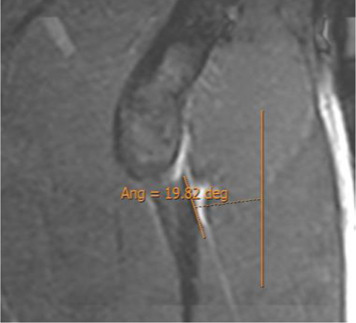 19.82°	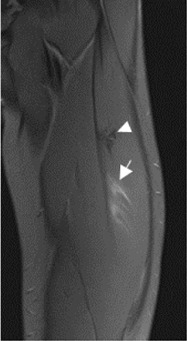
**Case C** common tendon tear (arrow)	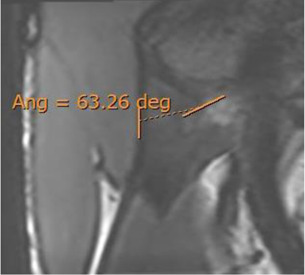 **63.26°**	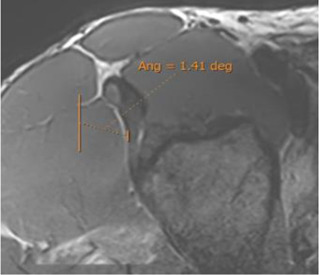 **−1.41°**	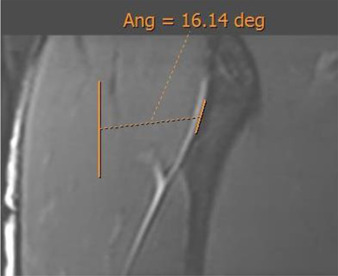 **16.14°**	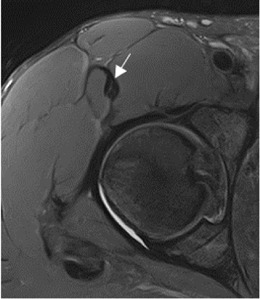

Angle DT-IT: double obliquity sagittal reconstruction, last slice where the DT line is drawn and the IT line is projected.

IT slope: oblique axial reconstruction and inclination of IT with respect to the anteroposterior axis.

DT slope: coronal reconstruction and inclination of DT with respect to the craniocaudal axis.

## Discussion

This study presented unprecedented radiological parameters to measure the angle between the DT and IT of the PTC of the RF. In addition, the inclination of these tendons in their respective craniocaudal and anteroposterior courses in the coronal and axial planes was also reported. The measurements were carried out in 112 MR scans of football players of different gender, sport discipline, and age.

The inter- and intra-observer agreement analyses in the proposed measurements of the radiological parameters (DT-IT angle, IT slope, and DT slope) have been evaluated through the B&A plot (Bland JM and Altman DG, 1999; Giavarina D, 2015). The B&A analysis recommended that the criteria for good agreement are that the mean difference of the measure between observers should be ∼0 and that 95% of the data points should lie within ± 2 s of the mean difference (Bland JM and Altman DG, 1999; Giavarina D, 2015). In all three radiological parameters, the B&A plot demonstrated inter-observer agreement (Figure 6). In the IT slope, the inter-observer agreement is where more outliers were found. It should be due to the wavy morphology and lateral convex margin of the IT. These characteristics make it difficult to decide which trajectory should follow the IT line to measure the inclination.

The intra-observer agreement B&A analysis for the different radiological measurements is presented in Figure 7. In the case of the angle DT-IT and the DT slope, there was intra-observer agreement. In the case of the IT slope measurement of observer 1, the intra-observer agreement B&A analysis showed that 0 is excluded from the confidence interval limits for mean difference (Figure 7B). When the plot of differences is shown as B&A plot difference percentage, 0 is included in the confidence interval limits for the mean difference. Therefore, the intra-observer agreement B&A analysis for the IT slope measure of observer 1 meets criteria B&A. Differences in the intra-observer agreement between observers could be due to experience. The more experienced observer (observer 1) could, at some point, draw a line between some parameters following the instinct that develops with their own experience. Conversely, in the case of the less experienced observer (observer 2), they probably follow the rules of the measurement method with accuracy.

The radiological measurements of the PTC proposed by us (DT-IT angle, IT slope, and DT slope) showed inter- and intra-observer agreement, although the IT slope measurement showed a slightly lower degree of inter- and intra-observer agreement according to B&A analysis, which could be due to the IT wavy morphology and its convex lateral margin. Conversely, the DT slope measurement showed the highest inter- and intra-observer agreement according to B&A analysis, probably due to its well-defined margins in most cases. Furthermore, according to the ICC results, the inter- and intra-observer reliability for measuring the radiological parameters was excellent.

In addition, population variability was found in all our measurements, and neither the DT-IT angle nor the tendon inclinations was correlated with gender, dominant limb, examined limb, or football discipline. In the case of the age range, there was no correlation between the DT-IT angle and the IT inclination, but the DT slope showed a correlation with age, probably because the samples between both ranges are different. Another limitation of the study is the small sample size of bilateral studies to compare dominant and non-dominant limbs.

The PTC of the RF has been extensively studied. Atzmon et al. (2020) analyzed the IT in an anatomical study with human cadavers to assess its clinical applications in labral surgical reconstruction. The authors described different PTC variables, among which the angle between the longitudinal axis of the muscle belly of the RF and the free IT (mean 135.8°) and the angle between the free IT and its footprint are included (Atzmon R et al., 2020). The latter cannot be measured by MRI, given the poor definition of the craniocaudal margins of the footprint portion. The difference between the DT-IT angle described here and that measured by Atzmon et al. (2020) between the RF muscle belly and the free IT lies in the origin of the measurement; the line along the DT path was drawn, which follows an axis different from that of the muscular belly.

To draw the IT line, the free tendon portion was considered as Atzmon et al. (2020) did, because the insertional portion is a complex network difficult to define by MR. The IT in its free tendon portion can present cranial or caudal expansions. However, they can be clearly defined and do not hinder the correct definition of the superior margin of the tendon when measuring the angle. Other studies have also been conducted to evaluate the measurements of the insertional portions of the DT and IT, demonstrating that these attachments were located in a broad area of the anterolateral pelvis and close to critical neurovascular structures (Hapa O et al., 2013; Ryan JM et al., 2014). These neurovascular structures can be identified by MRI and are differentiated from the typical expansions of IT by the characteristic irregular and serpiginous path of vascular structures. Previous studies have described in detail the anatomy of the PTC in cadavers (Testut L, 1884; Paturet G, 1951; Hasselman CT et al., 1995), and some have used MRI techniques (Bordalo-Rodrigues M and Rosenberg ZS, 2005; Ouellette H et al., 2006; Gyftopoulos S et al., 2008; Kassarjian A et al., 2012). However, to the best of our knowledge, this work is the first study to assess the degree of the amplitude of the angle between the IT and the DT when they merge (in the CT), without being influenced by the complexity of the insertion of the IT or the DT. In addition, the inclination presented by the IT and DT has already been reported in their free tendon path up to their respective insertions in the axial and coronal planes.

In an ultrasound study of the IT anatomy, Moraux et al. (2015) assessed the thickness, length, and inclination of the IT in the axial plane according to the inclination of the transducer (29.7°). In agreement with our results, they found no statistical differences in tendon measurements and angulation of the IT between the left and right sides nor between men and women (Moraux A et al., 2015). Although Atzmon et al. (2020) suggested that musculoskeletal structures could evolve with age (Atzmon R et al., 2020), their results did not show any statistical differences according to that parameter. In addition, in this study, no statistically significant differences were found between the two studied different football disciplines (association football and futsal) or between lateral dominance, suggesting no adaptive variation of the PTC morphology to the sporting activity.

The most frequent injuries in athletes are muscle injuries, and, in the case of football and its variants, 19% are myoconnective injuries of the RF (Ekstrand K et al., 2011; Isern-Kebschull J et al., 2020). Several studies have monitored these injuries in Australian and Spanish football players and have shown that they usually represent a long time of rehabilitation (Mendiguchia J et al., 2013), particularly if they present three out of the following four radiological signs: proximal location, injury seen in T1 weighted images, cross-sectional area >50%, and intermuscular fluid (Rodrigo RM, et al., presented at the 2009 annual meeting of the American Roentgen Ray Society) (Kassarjian A et al., 2012). The most frequent RF lesion involves the central septum, which belongs to the proximal MTJ and was first described by Hughes et al*.* (Hughes C et al., 1995; Balius R et al., 2009; Mendiguchia J et al., 2013; Mariluis and CupitoMamone, 2015).

The results demonstrated a population variability in the degree of opening of the DT-IT angle and the inclination of these tendons and that this variability did not depend on demographic factors, except for the DT slope and the older age group. This study is a prelude to the correlation between radiological parameters and the risk of injury. After we have demonstrated the reproducibility of the radiological parameters and their utility in assessing a PTC, the next step will be to validate a screening program. Following the process of development and validation of a screening program, where Bahr recommends three steps (Bahr R, 2016; Lee Dow C et al., 2021) to analyze the predictive capacity of the radiological parameters of the CTP as risk factors for injury, the cohort of men and soccer players should be selected. In the future, once we achieve an adequate sample size to consolidate the results, we will be able to demonstrate the correlation between the parameters and the risk of injury. We can only suggest the possibility of an anatomical pattern that may constitute a risk factor for injury in the proximal MTJ, markedly open DT-IT angle, and the rectification of the inclinations of the IT and DT, independently (Table 7, Case B) or in combination (Table 7, Case C).

Detecting early anatomical patterns that can exert biomechanical effects that play an important role in the pathogenesis of myoconnective lesions of the proximal MTJ of the RF should be one of the main challenges for sports medicine departments. This, in turn, will foster the establishment of more individualized preventive programs.

The main limitation of this study was the relatively small sample size. This precluded the evaluation of possible anatomical predisposition to RF injury based on the degree of opening of the DT-IT angle and the inclination of these tendons.

In conclusion, this study presented and analyzed unprecedented radiological parameters to measure the DT-IT angle and the IT and DT slope to further characterize the elements forming the RF. All measurements showed inter- and intra-observer agreement and anatomical variability of the PTC unrelated to the demographic factors of the population. These results allow going a step further in the detection of possible morphological patterns that represent a risk factor for lesions in the proximal MTJ of the RF in professional football and futsal players, extending the analysis to other sports disciplines.

## Data Availability

The raw data supporting the conclusion of this article will be made available by the authors, without undue reservation.
